# Anthocyanin Effects on Vascular and Endothelial Health: Evidence from Clinical Trials and Role of Gut Microbiota Metabolites

**DOI:** 10.3390/antiox12091773

**Published:** 2023-09-18

**Authors:** Samuele Laudani, Justyna Godos, Federica Martina Di Domenico, Ignazio Barbagallo, Cinzia Lucia Randazzo, Gian Marco Leggio, Fabio Galvano, Giuseppe Grosso

**Affiliations:** 1 Department of Biomedical and Biotechnological Sciences, University of Catania, 95123 Catania, Italy; s.laudani91@hotmail.it (S.L.); federica.didomenico@phd.unict.it (F.M.D.D.); ignazio.barbagallo@unict.it (I.B.); gianmarco.leggio@unict.it (G.M.L.); fgalvano@unict.it (F.G.); giuseppe.grosso@unict.it (G.G.); 2 Department of Agriculture, Food and Environment, University of Catania, 95123 Catania, Italy; cranda@unict.it; 3 ProBioEtna, Spin-Off of University of Catania, 95123 Catania, Italy

**Keywords:** anthocyanins, cardiovascular disease, microbiota, hypertension, polyphenols

## Abstract

Hypertension and derived cardiovascular disease (CVD) are among the leading causes of death worldwide. Increased oxidative stress and inflammatory state are involved in different alterations in endothelial functions that contribute to the onset of CVD. Polyphenols, and in particular anthocyanins, have aroused great interest for their antioxidant effects and their cardioprotective role. However, anthocyanins are rarely detected in blood serum because they are primarily metabolized by the gut microbiota. This review presents studies published to date that report the main results from clinical studies on the cardioprotective effects of anthocyanins and the role of the gut microbiota in the metabolism and bioavailability of anthocyanins and their influence on the composition of the microbiota. Even if it seems that anthocyanins have a significant effect on vascular health, more studies are required to better clarify which molecules and doses show vascular benefits without forgetting the crucial role of the microbiota.

## 1. Introduction

Hypertension, also known as high blood pressure (BP), is an important public health issue affecting approximately 1 billion people globally [1]. Hypertension is a leading risk factor for cardiovascular disease (CVD) and a major contributor to the burden of disease worldwide [2]. Elevated BP has been estimated to be responsible for approximately 7.5 million deaths and 127.5 million disability-adjusted life years each year [3]. In addition to its well-established link to heart disease and stroke, hypertension has also been linked to an increased risk of kidney disease, vision loss, and cognitive decline [4]. A better understanding of the pathogenic mechanisms and risk factors, as well as effective strategies for prevention and management, is critical for improving the health outcomes of individuals with hypertension. Genetics, environmental factors, and lifestyle factors, such as diet, play a role in the development of hypertension [5]. Diet, in particular, has a significant impact on BP regulation: a diet high in salt, saturated and trans fats, and added sugars has been shown to increase the risk of developing hypertension [6], while consumption of fruits, vegetables, whole grains, and lean proteins is under investigation for potential beneficial effects [7]. Fruits and vegetables are rich sources of various bioactive compounds, including anthocyanins. These compounds are (poly)phenols belonging to the family of flavonoids responsible for the red, purple, blue, or black colors of many fruits and berry fruits, such as grapes, but also black beans and rice, red cabbage, and blood oranges [8]. Anthocyanins are widely distributed in the plant kingdom and have a variety of properties, including antioxidant effects in plants [9]. A growing body of research suggests that anthocyanins may have a potential role in preventing CVD [10,11] and reducing the risk of hypertension [12]. Emerging evidence suggests that these compounds are largely metabolized by the gut microbiota and, subsequently, their effects on cardiovascular health would eventually be mediated by their metabolites [13]. However, the majority of preclinical studies focus on the putative antioxidant effects of anthocyanin per se, while a summary of metabolite formation operated by the gut microbiota and their actual role on cardiovascular health has not been reviewed yet. Thus, the aim of this article is to summarize the existing clinical evidence regarding anthocyanin supplementation’s effects on BP and various parameters of endothelial health, to review mechanistic studies on the role of gut microbiota on anthocyanin metabolite production, and to discuss the implications for the use of anthocyanin supplementation as an intervention for the prevention of hypertension.

## 2. Markers of Vascular and Endothelial Function

BP is a primary risk factor for endothelial dysfunction [14]. Reactive oxygen species (ROS) are continuously generated at low concentrations in endothelial cells due to transitory hyper- and hypoglycemia, hypoxia, and ischemia/reperfusion further contributing to the pro-oxidative and proinflammatory processes.

**Figure 1:**
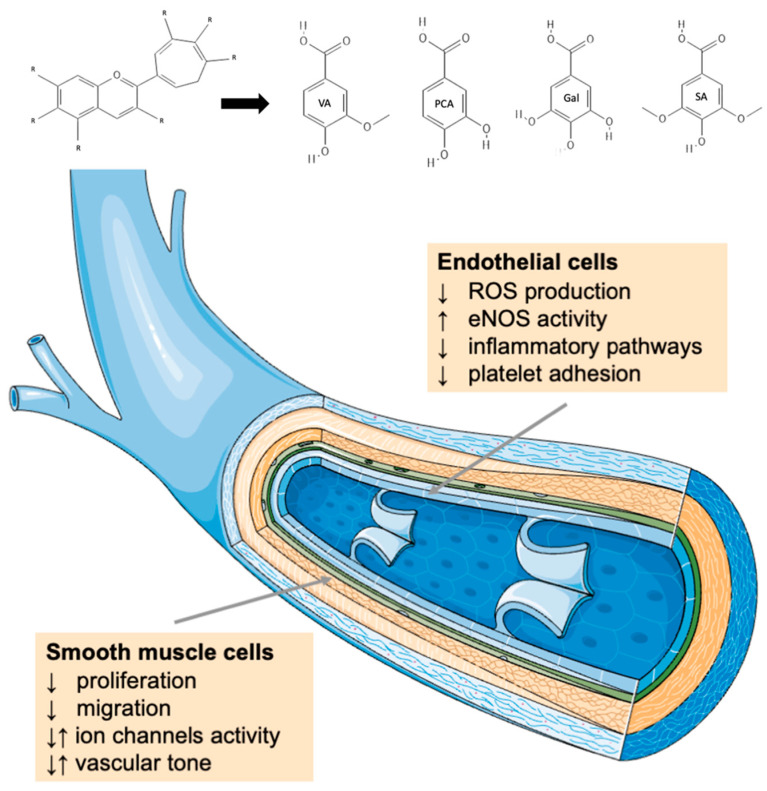
Main pathways through which anthocyanins inhibit oxidative stress and inflammatory processes, including reactive oxygen species scavenging and inhibition of inflammatory cascades resulting in decreased expression of adhesion molecules and inhibition of platelet adhesion. Abbreviations: Gal (gallic acid); PCA (protocatechuic acid); SA (syringic acid); VA (vanillic acid).

These conditions lead to the production of inflammatory cytokines, such as interleukin (IL)-1 beta, tumor necrosis factor-alpha (TNF-α), and C-reactive protein [15]. These molecules bind to their receptors and trigger the inflammatory signaling pathways involving protein kinase B (PKB/Akt), transcription factor nuclear factor-kappa B (NF-κB), mitogen-activated protein kinase p38, and extracellular signal-regulated kinases (ERK)1/2 leading to activation of cell adhesion molecules, a significant group of endothelial markers important for maintaining the integrity of the blood vessels [16]. Other markers influenced by the inflammatory state are proteins involved in nitric oxide (NO) synthesis and molecular adhesion. NO is a gas molecule with great importance in cardiovascular health and disease. NO could be synthesized via three isoforms of nitric oxide synthase (NOS), namely inducible NOS, endothelial NOS (eNOS), and neuronal NOS, and the correct balance among these enzymes is essential for cardiovascular health [17]. In fact, endothelial dysfunction leads to an abnormal pro-inflammatory and prothrombotic phenotype of the endothelial cells lining the lumen of blood vessels, unbalancing NO bioavailability, impairing vascular tone, and ultimately causing vascular damage [18]. Adhesion molecules, such as intercellular adhesion molecule-1 (ICAM-1) and vascular cell adhesion protein-1 (VCAM-1), are constitutively present on endothelial cells. Inflammatory processes triggered by different factors can modify their expression, facilitating transmigration of leukocytes from blood vessels into tissues and contributing to the pathogenesis of cardiovascular conditions underlined by systemic oxidative stress and inflammation [19,20]. Anthocyanins are poorly absorbed in the gastrointestinal tract and are mainly metabolized by the gut bacteria in other molecules such as gallic acid (Gal), protocatechuic acid (PCA), syringic acid (SA), and vanillic acid (VA) [21]. Many studies have explored the antioxidant and anti-inflammatory effects of anthocyanins and their metabolites [22,23], demonstrating their capacity to regulate NO production and exhibit scavenger activity, thus reducing ROS concentration and production [24] (Figure 1).

## 3. Clinical Studies on Anthocyanin-Containing Whole Fruits

A summary of randomized clinical trials (RCTs) providing anthocyanin doses for interventions in endothelial and vascular outcomes is presented in Table 1. Only one study was conducted using whole fruits on healthy volunteers. A double-blind crossover RCT was carried out to explore the potential benefits of anthocyanins on endothelial function in 14 older participants, who were divided into the treatment group (which received 600 mg of New Zealand blackcurrant containing 210 mg anthocyanins daily) and the placebo group for seven days, followed by crossover to the other treatment, separated by 4 weeks of washout. At the end of the study, carotid–femoral pulse wave velocity (PWV) (*p* = 0.03) and central BP (*p* = 0.02) were significantly reduced after the intervention, while brachial systolic blood pressure (SBP; *p* = 0.03), diastolic blood pressure (DBP; *p* = 0.02), mean (*p* = 0.01), and the augmentation index (*p* = 0.03) decreased significantly after blackcurrant intake compared with baseline values [25]. Similar studies conducted on individuals at high cardiovascular risk showed mixed findings. Among the few studies providing whole fruits as an intervention arm, a single-blind RCT conducted on 71 individuals at higher cardiovascular risk (mean age 58 y) administered two portions of berries daily (containing 515 mg/day of anthocyanins) for 8 weeks showed a significant decrease in mean SBP in subjects with high baseline (*p* = 0.024) compared to control. Moreover, a significant increase in the closing time on the platelet function analyzer when ADP and collagen were used as platelet activators was detected (*p* = 0.018) [26]. Another 8-week single-blind clinical trial investigated the effects of blueberry consumption (containing 154 mg/day anthocyanins) in 48 individuals with metabolic syndrome (mean age 50 y) and reported a significant reduction in SBP (*p* = 0.003) and DBP (*p* = 0.04) in the blueberry group (−6 and −4%, respectively) compared to the control group (−1.5 and −1.2%) [27]. A double-blind parallel RCT investigated the cardiovascular effects of the daily supplementation of one or one half cup of blueberries (containing 364 mg or 182 mg of anthocyanins respectively) in 115 individuals with metabolic syndrome (50–75 y): after 6 months of supplementation, a significant improvement in flow-mediated dilation (FMD) (1.45% vs. 0.39%; *p* = 0.003) and augmentation index (−2.24% vs. 0.24%; *p* = 0.04) was found [28]. In another crossover RCT investigating the potential action of the daily consumption of 250 mg of raspberries (containing 225 mg of anthocyanins) in 22 adults (mean age 54 y) with elevated waist circumference and diabetes, no significant differences were observed [29]. Similarly, another double-blind RCT investigated the vascular effects of the daily supplementation of 60 mL of Montmorency tart cherry (containing 73.6 mg of anthocyanins) in 23 healthy non-smoking volunteers and reported no significant differences in SBP and DBP after 4 weeks of consumption [30].

## 4. Clinical Studies on Anthocyanin-Containing Fruit Juices

Most studies investigating the potential effects of anthocyanins on vascular and endothelial health in healthy or low CVD-risk individuals have been performed by administering fruit juices. Among studies reporting significant findings, a crossover RCT investigating the effects of a 2-week oral treatment with Concord grape juice (CGJ) (containing 296 micromol/L of anthocyanins) on 26 healthy smokers on endothelial function, aortic stiffness, and BP showed that the treatment (7 cc/kg/d) led to a significant improvement in FMD (from 8.35 ± 2.83% to 9.49 ± 2.74%; *p* = 0.02) and PWV (from 6.13 ± 0.61 m/s to 5.63 ± 0.56 m/s; *p* = 0.04) in the CGJ group [31]. A double-blind parallel RCT conducted on 64 participants aimed to evaluate the effects of administration of 1000 mL per day of low-polyphenol content blackcurrant juice (40 mg/day anthocyanins), high-polyphenol content blackcurrant juice (142 mg/day anthocyanins), or placebo drink for 6 weeks, resulting in an increase in FMD (from 5.8 ± 3.1 to 6.9 ± 3.1%; *p* = 0.022) from the higher polyphenol content juice compared to placebo [32]. In another study conducted on 37 subjects (mean age of 55.8 years), it was observed that a daily intake of 1024 mg of anthocyanins from chokeberry (300 mL/day cold-pressed 100% chokeberry juice and 3 g/day oven-dried chokeberry powder) significantly decreased daytime ambulatory DBP (−1.64 mmHg *p* = 0.02) and tended to decrease 24-h DBP (−1.07 mmHg, *p* = 0.084) and the true awake ambulatory SBP (−2.71 mmHg, *p* = 0.077) and DBP (−1.62 mmHg, *p* = 0.057) compared to a placebo [33]. In another trial conducted on 112 healthy volunteers aged between 65 and 80 years, the effect of wild blueberry powder (500 mg and 1000 mg) or wild blueberry extract (111 mg) supplementation (which provided 1.35 mg/day, 2.7 mg/day and 7 mg/day of anthocyanins, respectively) or placebo for 6 months was evaluated: after treatment, the subjects administered the higher concentration reported a significant reduction in SBP compared to the placebo group (*p* = 0.039) [34]. A randomized controlled clinical trial evaluating the effect of administering 480 mL of tart cherry juice (450.6 mg/day of total phenolics and 95.9 mg/day total tannins) on BP showed that after 12 weeks of administration on a population of 34 participants (mean age of 72 years), a significant reduction of SBP in the treated group (from 141.4 ± 27.0 to 137.3 ± 5.6 mmHg; *p* = 0.04) compared to control group resulted [35]. Another single-blind crossover RCT was conducted on 15 healthy participants (with a mean age of 28.7 years) to evaluate the effects of 400 mL/day of blood orange juice (containing 960 mg anthocyanins): after 4 weeks, FMD increased significantly compared to the control drink (*p* = 0.001) [36].

In contrast to previous studies, an open-label, placebo-controlled trial conducted on 47 healthy adults with a mean age of 40 years showed that daily intake of 250 mL of cherry drink (providing 273.5 mg anthocyanins) did not lead to any significant change concerning BP or other endothelial parameters [37]. Another double-blind crossover RCT aiming to investigate the effects of CGJ (containing 167 mg of anthocyanidins and 334 mg of proanthocyanidins) in 19 healthy mothers (40–50 y) reported no effects on BP [38]. In a placebo-controlled single-blind crossover RCT, the effects of Montmorency tart cherry juice (MTCJ) on 11 healthy participants who consumed 260 mL/day of MTCJ (providing 540 mg of anthocyanins) or placebo for 20 days were evaluated, and no significant differences between conditions or interactions after the treatment were reported [39].

Among studies conducted in individuals at high CVD risk, 19 patients with an abnormal echo doppler of the carotids were separated to ingest either 50 mL/day of pomegranate juice (providing 19.2 mg of anthocyanins) or a placebo: after 1 year of treatment, common carotid intima-media thickness increased by 9% in the placebo group (from 1.52 ± 0.03 to 1.65 ± 0.04 mm; *p* < 0.01), whereas in the intervention group, it decreased by up to 30% (*p* < 0.01). An effect on carotid peak systolic velocity (*p* < 0.01), on end diastolic velocity (*p* < 0.01), and a decrease in SBP by 21% (*p* < 0.05) were also in evidence [40]. A small trial was conducted on 27 individuals with metabolic syndrome (mean age 47 y) who were recruited to investigate the effects of a strawberry beverage containing 154 mg of anthocyanins vs. a control group consuming 2 cups of water daily for 8 weeks resulted in a significant decrease of about 18% in VCAM-1 levels (from 272.7 ± 17.4 to 223.0 ± 14.0 ng/mL, *p* < 0.05) compared to the control group, but no significant changes were reported among the groups [41]. A double-blind crossover RCT compared the vascular effects of 4-week consumption of 480 mL double-strength cranberry juice per day (daily amount of anthocyanins 94 mg) vs. 480 mL of a placebo beverage per day in 44 overweight, middle-aged individuals with a high prevalence of cardiovascular risk factors resulting in mainly null effect on vascular parameters considered (brachial artery FMD, digital pulse amplitude tonometry, and carotid-radial PWV) but decreased carotid-femoral PWV by 0.5 m/s (6%) after cranberry juice consumption, while it increased by 0.4 m/s after placebo consumption (*p* = 0.003) [42]. In another study, 35 sedentary overweight men at high CVD risk were supplemented with 500 mL of cranberry juice (providing 20.8 mg of anthocyanins) or placebo juice per day for 4 weeks, followed by 4 weeks of washout before crossing. Significant differences were observed, showing a decrease in augmentation index, index of arterial stiffness, (from 19.8 ± 9.7 to 17.8 ± 10.9%; *p* = 0.027), and global endothelial function (from 1.02 ± 0.56 to 0.42 ± 1.93; *p* = 0.02) after the intervention [43]. Another double-blind RCT conducted on 36 participants with type-2 diabetes (mean age 51 y) investigated the effects of a freeze-dried strawberry drink (containing 154 mg of anthocyanins) versus placebo. After 6 weeks of administration, results showed a significant reduction in DBP in the intervention group compared to placebo (from 84.2 ± 8.03 to 78.7 ± 7.2 mmHg, *p* = 0.014) [44]. In a single-blind RCT in which 21 hypertensive participants were assigned to receive either 150 mL/day of pomegranate juice (containing 8.7 mg anthocyanins) or water for 2 weeks, the results were a significant reduction in SBP (from 130.91 ± 13 to 124.55 ± 15.72 mmHg; *p* = 0.008) and DBP (from 80 ± 8.94 to 76.36 ± 6.74 mmHg; *p* = 0.046) and a significant increase in FMD (%) (from 0.23 ± 0.09 to 0.29 ± 0.07; *p* = 0.034) in the intervention group compared to baseline; moreover, a significant reduction in VCAM-1 (*p* = 0.008) and a significant increase in E-selectin (*p* = 0.039) was reported [45]. In a double-blind RCT conducted for 12 weeks in 130 healthy individuals aged 50–70 years with high normal range BP (130/85–139/89 mmHg) or stage 1–2 hypertension (140/90–179/109 mmHg) were administered either 500 mL/day of a commercially available juice based on red grapes, cherries, chokeberries, and bilberries (anthocyanin content of 11.9 mg/100 g), an anthocyanin-enriched juice with polyphenol-rich extracts from blackcurrant press-residue (anthocyanin content 41.3 mg/100 g), or a placebo juice led to a significant decrease of SBP over time (6 and 12 weeks, respectively) in the intervention juice groups compared with the placebo group (−6.9 and −3.4 mmHg, respectively; *p* = 0.01), with more pronounced effects in hypertensive subjects (−7.3 and −6.8 mmHg, respectively; *p* = 0.04) [46]. Another study conducted on 42 adults aged 70 years or older with mild-to-moderate dementia investigated the effects of 200 mL/day of cherry juice (anthocyanins daily content: 138 mg) or 200 mL/day of commercially prepared apple juice led to a significant reduction in SBP (*p* = 0.038) at 6 and 12 weeks post-baseline (SBP at baseline: 138.2 ± 16.4 mmHg, 6 weeks: 133.7 ± 9.9 mmHg, 12 weeks: 130.5 ± 12.2 mmHg) and a similar trend for DBP, albeit not significant (*p* = 0.160) [47]. In a double-blind crossover RCT, the effect of 500 mL of pure pomegranate juice (containing 50.26 mg of anthocyanins) on 30 participants (mean age 51 y) with metabolic syndrome compared to a placebo group showed that the intervention was more effective in reducing SBP (from 139.43 ± 2.29 to 131.73 ± 73 mmHg, *p* < 0.0001) and DBP (from 92.7 ± 1.85 to 87.80 ± 1.40 mmHg; *p* = 0.02) than the control [48].

Otherwise, various studies did not find any significant difference between the treatment and placebo group among individuals at high CVD risk. In a double-blind parallel RCT, 32 obese, non-diabetic, and insulin-resistant individuals were supplemented with a smoothie of blueberries containing 668 mg of anthocyanins or with a placebo smoothie for 8 weeks; at the end of the treatment, no significant changes in BP were found [49]. A double-blind RCT was conducted to investigate the role of 480 mL/day cranberry juice supplementation (containing 24.8 mg of anthocyanins) on vascular health in 36 individuals (mean age 52 y) with metabolic syndrome; after 8 weeks of cranberry juice intervention, the authors reported a lack of significant findings in BP compared to baseline values [50]. Similar results were obtained from a double-blind RCT in which 21 participants (76 years) with mild age-related memory decline were supplemented with grape juice or placebo for 16 weeks and which reported no significant change in BP compared to placebo [51]. In another study, 69 participants (mean age 49 y) with peripheral endothelial dysfunction and cardiovascular risk factors were supplemented with 460 mL of cranberry juice (containing 69.46 mg anthocyanins, 1224.52 mg proanthocyanidins) or 460 mL of placebo per day for 4 months; results showed a significant decrease in osteoblastic marker osteocalcin positive endothelial progenitor cells (−8.64 ± 48.98 as compared to 19.13 ± 46.11, *p* = 0.019) [52]. In another study, 60 volunteers with abdominal adiposity and elevated serum lipids were supplemented with high-dose freeze-dried strawberry beverages (containing 115 mg of anthocyanins), low-dose freeze-dried strawberry beverages (containing 78 mg of anthocyanins), or a control beverage for 12 weeks, but no significant findings were noted in SBD and DBP between groups [53]. Similar results have been observed in a double-blind, placebo-controlled, parallel clinical trial that investigated the effects of 480 mL/day low-calorie cranberry juice consumption (containing 236 mg of proanthocyanidins) in 56 obese volunteers (25–65 y); after 8 weeks, DBP decreased significantly in the low-calorie cranberry juice group compared to the placebo beverage (69.2 ± 0.8 vs. 71.6 ± 0.8 mmHg; *p* = 0.048) [54]. Another study including 19 adults at high risk of type-2 diabetes mellitus (mean age 53 y) who received 240 mL of wild blueberry juice daily, containing 314 mg of anthocyanins, or a placebo beverage showed no significant results, but an increase of nitrite and nitrate, a NO index product, was observed after treatment (from 2.9 ± 0.4 μM to 4.1 ± 0.4 μM; *p* = 0.039) [55]. Additionally, another RCT involving 23 women (40–60 y) with metabolic syndrome investigated the effects of 300 mL pomegranate juice daily (containing 6.3 mg of anthocyanins) for 6 weeks showed that although not significantly, the consumption of pomegranate juice determined a tendency to decrease SBP [56]. An open-label, two-arm crossover RCT evaluated the effects of 500 mL/day of blood orange juice (containing 50 mg of anthocyanins) compared to blonde orange juice without anthocyanins on 41 obese participants treated for 4 weeks, with no significant differences observed between groups [57]. A double-blind crossover RCT investigated the effects of the daily consumption of 200 mL of agraz nectar, containing 4.66 mg of anthocyanins, on 40 women (28–66 y) with metabolic syndrome showed that both SBP and DBP were not affected by the treatment [58]. In a double-blind parallel RCT, the effect of chokeberry juice supplementation with different doses of anthocyanins (113.3 mg cyanidin-3-glucoside; 28.3 mg cyanidin-3-glucoside) on 80 adults (mean age 40 y) at CVD risk for 4 weeks was evaluated; however, no differences were observed after the treatment period [59]. Another single-blind parallel RCT was carried out to investigate the endothelial effects of 12-week supplementation with 480 mL/day of tart cherry juice (containing 176 mg of anthocyanins) in 26 individuals (20–40 y) with metabolic syndrome reported that at the end of the trial, no significant changes were reported in BP [60].

## 5. Clinical Studies on Anthocyanin-Containing Extracts

Some studies conducted on healthy or low CVD-risk subjects administered extracts from grapes and other fruits. In a parallel RCT involving 43 non-smoking adults (with a mean age of 35 years), researchers administered 7.5 g grape antioxidant dietary fiber (providing about 60 mg of anthocyanins) daily for 16 weeks and reported a significant decrease in SBP (from 126.5 ± 22.1 to 118.0 ± 19.6 mmHg; *p* < 0.05) and DBP (from 78.2 ± 11.7 to 74.4 ± 12.1 mmHg; *p* < 0.05) in the intervention group compared to baseline [61]. Similar results were observed on 70 healthy men and postmenopausal women, aged between 35 and 75 years, who underwent to a significant reduction of SBP (from 135 to 130 mmHg; *p* < 0.01) and DBP (from 81.9 to 79.1 mmHg; *p* < 0.01) compared to placebo group after administration of 300 mg of grape seed extract [providing monomeric procyanidins (4.3%), dimeric procyanidins (6.1%), trimeric procyanidins (2.5%), Gal (4.8%)] for 8 weeks [62]. A double-blind RCT involved 91 women (40–60 y) with at least one menopausal symptom each, who received grape seed extract tablets containing different doses of proanthocyanidins (100 mg/day or 200 mg/day) or placebo for 8 weeks; at the end of the trial, a significant reduction in SBP and DBP was detected in both low- and high-dose polyphenol groups (*p* < 0.001) [63]. Finally, two studies investigated the effects of *Aronia melanocarpa* extract (30 mg/day anthocyanins) after 12 [64] or 24 weeks (27 mg/day anthocyanins) [65] resulting in a significant increase in FMD and a significant reduction in brachial DBP, respectively.

In contrast, in an earlier double-blind four-armed parallel RCT, 69 healthy participants (38–74 y) were administered either (i) red wine (males: 300 mL/day, containing 87 mg of anthocyanins; females: 200 mL/day, containing 58 mg of anthocyanins), (ii) water and red grape extract tablets (wine-equivalent dose in terms of polyphenols: M: 71 mg anthocyanins; F: 48 mg anthocyanins), (iii) water and red grape extract tablets (half dose of polyphenols: M: 36 mg anthocyanins; F: 24 mg anthocyanins), or (iv) water and placebo tablets for 4 weeks. At the end of the study, no significant differences in SBP or DBP were observed among the groups [66]. Similarly, a double-blind crossover RCT examined the effects of 300 mg/day New Zealand blackcurrant extract (containing 105 mg anthocyanins) or a placebo in 14 healthy trained cyclists showed no differences in BP after 7 days of supplementation [67]. In a double-blind crossover RCT conducted on 17 participants (mean age 57 years) with osteoarthritis, the administration of 100 g/day of strawberry powder (providing 132 mg total anthocyanins, 440 mg ellagic acid, 100 mg phytosterols) for 26 weeks followed by crossover resulted in no differences in BP compared to control treatment [68]. More recently, another double-blind RCT aimed to investigate the effects of 3 months’ consumption of 60 mL/day of tart Montmorency cherry extract (containing 68–73.5 mg of anthocyanins) in 23 adults (mean age 23 y) reported no significant changes observed in BP and arterial stiffness between the groups [69]. In another randomized repeated measures crossover study conducted on 18 males, the administration of a wild blueberry drink for 6 weeks, which provided 375 mg/day of anthocyanins, did not show any significant changes in endothelial function compared to placebo [70]. Finally, a double-blind crossover RCT compared the effects of the daily supplementation of 320 mg of anthocyanins and placebo capsules in 16 healthy individuals (mean age 38 y); no significant findings were reported in BP and pulse [71]. 

Other studies used extracts and powder in individuals at high CVD risk. An earlier double-blind RCT tested the effects of 6 weeks of 85 mg of chokeberry flavonoid extract (containing 21 mg of anthocyanins) administered three times a day in 44 patients who had survived myocardial infarction and were under statin therapy (mean age of 66 y), which resulted in improvements in SBP and DBP compared to baseline levels by a mean average of 11 mmHg (*p* < 0.001) and 7.2 mmHg (*p* < 0.001), respectively, and lowering of the level of adhesion molecules VCAM, ICAM and monocyte-chemoattractant molecule 1 (MCP-1) while increasing adiponectin concentration as compared with placebo [72]. Significant findings have been obtained from a crossover study conducted on 24 men (mean age 50 y) with metabolic syndrome, which investigated the effects of grape polyphenols (46 g/day of grape powder containing 35.42 mg anthocyanins) on cardiovascular risk factors; after 30 days of treatment with 3 weeks of washout and crossover, the treatment led to a significant reduction in SBP (122 ± 11 mmHg of grape group vs. 128 ± 10 mmHg of placebo group; *p* < 0.025), a significant increase in FMD (5.7% ± 2.96% and 0.28 ± 0.15 mm in grape group versus 4.0% ± 2.4% and 0.20 ± 0.12 mm in the placebo group; *p* < 0.001), and a significant reduction in soluble intercellular CAM-1 concentration (*p* < 0.025) compared to the placebo [73]. Another double-blind RCT explored the effects of 22 g freeze-dried blueberry powder per day (providing 103.18 mg/day of anthocyanins) or 22 g control powder containing maltodextrin on 40 postmenopausal women with pre- and stage 1- hypertension for 8 weeks; at the end of the intervention, both SBP and DBP (131 ± 17 mmHg; *p* < 0.05 and 75 ± 9 mmHg; *p* < 0.01, respectively) and brachial-ankle PWV (1401 ± 122 cm/second; *p* < 0.01) were significantly lower than baseline levels in the intervention group [74]. Another study involving 44 adults with metabolic syndrome showed that 45 g/day of blueberry smoothie with freeze-dried blueberry powder (containing 580.6 mg anthocyanins) consumption did not differ from a placebo group in terms of BP and endothelial function; however, the mean change in resting endothelial function, expressed as reactive hyperemia index, was significantly more improved in the intervention group than in the placebo one (0.23 ± 0.14 vs. −0.23 ± 0.13; *p* = 0.024) [75]. In a double-blind parallel RCT, 60 postmenopausal women aged between 45 to 65 years with pre- and stage 1-hypertension were divided into three groups and supplemented with 25 g/day (containing 102.13 mg of anthocyanins) or 50 g/day (containing 204.26 mg of anthocyanins) of freeze-dried strawberry powder or 50 g/day of placebo for 8 weeks; a reduction of SBP, brachial- and femoral-ankle PWV (141 ± 3 to 135 ± 3 mmHg, *p* = 0.02; 15.5 ± 0.5 to 14.8 ± 0.4 m/s, *p* = 0.03, and 11.0 ± 0.2 to 10.4 ± 0.2 m/s, *p* = 0.02, respectively) ensued in the 25 g/day intervention arm, but no significant changes were apparent in the other arms [76]. An open-label RCT tested daily bilberry powder ingestion (containing 900 mg of anthocyanins) in 50 subjects with post-myocardial infarction (median age 68 y); after 8 weeks, non-significant reductions in SBP, DBP, and heart rate were reported in the bilberry group compared to the placebo group [77]. 

## 6. Clinical Studies on Anthocyanins Administered in Capsules/Tablets

A group of studies also administered anthocyanin extracts via capsules or tablets. Among larger studies, 146 hypercholesterolemic participants, aged 40–65 years, were treated with 4 anthocyanin capsules providing a total daily intake of 320 mg/day anthocyanins or placebo for 12 weeks, resulting in a significant decrease in SBP (from 126.2 ± 14.9 to 119.5 ± 12.5 mmHg, *p* < 0.05) and a significant increase in FMD (from 8.04 ± 1.82 to 10.91 ± 2.06%, *p* < 0.05) compared to baseline [78]. A similar double-blind RCT involving 51 individuals with metabolic syndrome who were treated with 4 capsules/day (equivalent to 750 mg/day of black raspberry and providing 0.75 mg cyanidin, 0.28 mg of catechin, 0.04 mg of epicatechin, 0.03 mg of quercetin, and 0.15 mg proanthocyanidins) showed a significant reduction in radial artery augmentation index was observed in the black raspberry group compared to the placebo group (−5% ± 10% vs. 3% ± 14%; *p* < 0.05) after a 12-week intervention [79]. However, other studies providing similar interventions reported null results. A clinical trial conducted among 52 healthy postmenopausal women with a mean age of 58 years investigated the effects of the daily consumption of 4 capsules of elderberry extract providing about 500 mg of anthocyanins compared to a placebo for 12 weeks led to no significant differences in BP and pulse rate [80]. Similar null results have been observed in a double-blind crossover RCT study in which the effects of anthocyanin capsules supplementation (providing 640 mg of anthocyanins) in 31 healthy men for 4 weeks were investigated [81]. A double-blind RCT was conducted in 42 overweight smokers (45–65 y) consuming either a standardized extract of maqui berry (providing 162 mg anthocyanins) or maltodextrin (placebo) capsule 3 times daily for 4 weeks showed no significant differences in BP levels after the treatment [82]. In another double-blind RCT, 146 hypercholesterolemic subjects aged between 44 and 65 years were assigned to consume 4 capsules containing 320 mg of anthocyanins or 4 placebo capsules daily: after 24 weeks, no significant results were observed in BP among the different groups [83]. In a parallel RCT, 72 postmenopausal women were supplemented with 60 mg/day of anthocyanins, 6 mg lutein +2 mg zeaxanthin/day, or a combination of both, in the form of capsule, for 8 months; at the end of the study, no differences were observed among groups [84].

## 7. Anthocyanins and Their Gut-Microbiota Derived Metabolites on CVD

**Figure 2:**
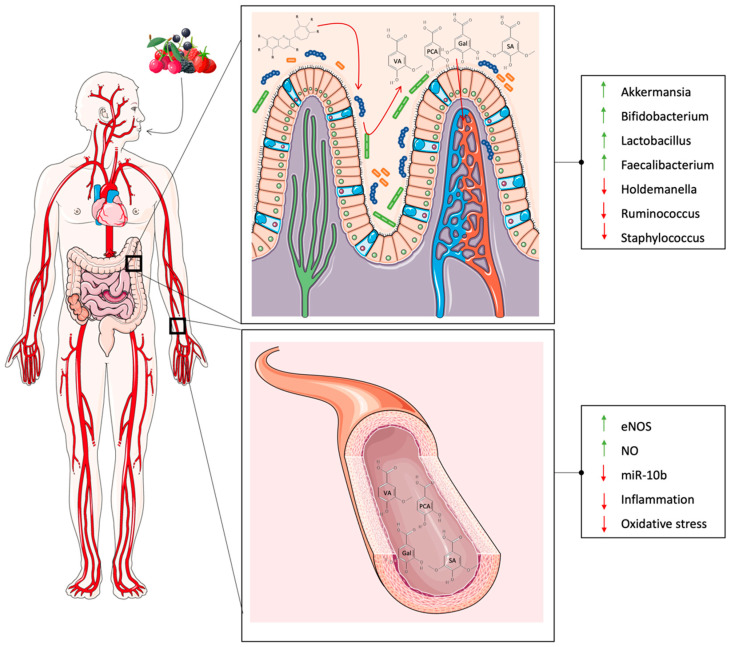
Effects of anthocyanins’ derived metabolite on microbiota and cardiovascular system. Anthocyanins are metabolized by bacteria inhabiting the gut, producing different metabolites which exert their effect on the microbiota composition and on the cardiovascular system leading to an increase of beneficial bacteria and anti-inflammatory markers. Abbreviations: Gal (gallic acid); PCA (protocatechuic acid); SA (syringic acid); VA (vanillic acid).

The human gut is inhabited by trillions of microorganisms responsible for digesting a variety of compounds in the diet [85]. Due to the advances of the omics approach, such as sequencing technologies and bioinformatics, the gut microbial community has been linked to various diseases, including CVD. In particular, microbial community composition has garnered research interest due to the production of several metabolites that could exert positive or negative effects on human health. Several studies have investigated the possible relationship between microbiota and CVD (Figure 2).

**Figure 3:**
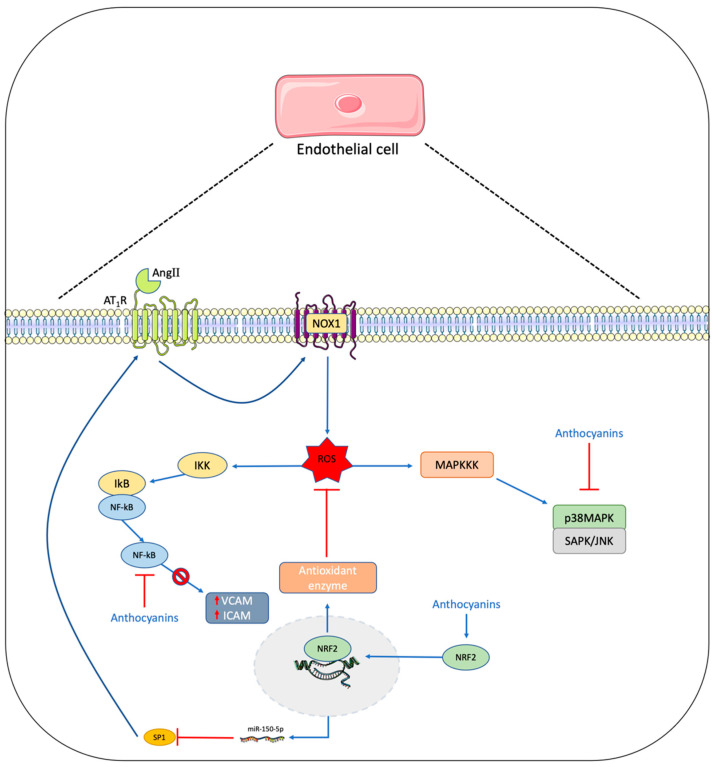
The main mechanisms through which anthocyanins can influence the expression of proinflammatory and antioxidant proteins. Anthocyanins can block NF-κB, reducing the expression of VCAM and ICAM proteins, and can meanwhile activate NRF2-inducing antioxidant enzyme production and regulate miR-150 levels. Abbreviations: ATR (angiotensin II type-I receptor), AngII (angiotensin II), ICAM (intercellular adhesion molecule-1), IKB (inhibitor of nuclear factor-kappa B), IKK (IKB kinase), JNK (c-Jun N-terminal kinases), MAPKKK (MAPK kinase kinase), NF-κB (nuclear factor-kappa B), NOX1 (NADPH oxidase 1), NRF2 (nuclear factor erythroid 2-related factor 2), p38MAPK (MAP-kinase p38), ROS (reactive oxygen species), SAPK (stress-activated protein kinases), SP1 (specific protein 1), VCAM (vascular cell adhesion protein-1).

In one cohort study, hypertensive and pre-hypertensive participants showed a significant increase of *Prevotella* and *Klebsiella* genera and a significant reduction of *Faecalibacterium*, *Oscillibacter*, *Roseburia*, *Bifidobacterium*, *Coprococcus*, and *Butyrivibrio* compared to healthy participants [86]. Other studies reported microbiota alterations in patients with heart failure, with decreased levels of the *Faecalibacterium prausnitzii* and Lachnospiraceae family and increased levels of *Ruminococcus*, *Prevotella*, *Hungatella*, and *Succinclasticum* genera compared to controls [87]. In addition, in early studies, gut microbial compositional changes have been discovered in patients with CVD risk factors, including hypertension, insulin resistance, dyslipidemia, and other metabolic phenotypes. It has already been established that gut microbial community is involved in anthocyanin metabolism, and their metabolites have a significant impact on cardiovascular health, both directly and indirectly, via the modulation of gut microbiota composition. Anthocyanin metabolism regulates the growth of specific bacteria in the gut, promoting the proliferation of healthy anaerobic bacteria. The main bacterial groups that can metabolize anthocyanins are the health-promoting genera *Bifidobacterium* and *Lactobacillus*. In vivo studies conducted on high-fat diet mice have shown that anthocyanin-rich berry extracts increased the genera *Bifidobacterium*, *Lactobacillus*, *Roseburia*, *Faecalibaculum*, and *Parabacteroides*, while reducing the genera *Staphylococcus* [88] *Allobaculum*, *Anaerotruncus*, *Intestinimonas*, *Oscillibacter* and *Ruminiclostridium* [89]. Additionally, F-344 rats supplemented with black raspberries exhibited a significant change in microbiota composition with a significant increase in *Akkermansia* and *Desulfovibrio* genera, known for their anti-inflammatory properties, and the *Anaerostipes* genus, which is a butyrate-producing bacteria [90]. Anthocyanin intake has led a significant reduction in *Clostridium* abundance and an increase in *Barnesiella* [91] as well as *Clostridium XVIa*, *Roseburia*, and *Prevotella* [92] in a mouse model. The reduction of *Clostridium* is generally considered beneficial, but *Clostridium XVIa* does not produce toxins or virulence factors and may inhibit macrophage infiltration in adipose and hepatic tissues [13]. The beneficial effects of berry intake and the consequent introduction of polyphenols can be correlated with the creation of a redox environment promoting the growth of strict anaerobes, such as *Bacteroidetes* and *Actinobacteria* populations [93]. Findings from in vitro and animal studies have shown that anthocyanins can regulate the expression of different genes involved in many cellular processes [94] (Figure 3).

Anthocyanins have been observed to downregulate the expression of cell adhesion molecules such as E-selectin, VCAM-1, and ICAM-1 as well as genes involved in inflammatory response, such as IL-8 and MCP-1, and the NF-κB [95]. Furthermore, anthocyanins trigger eNOS phosphorylation, thereby increasing NO production [96], and are involved in the reduction of ROS production induced via endothelial cell activation [9]. Anthocyanin metabolites, such as Gal, 3-O-methylgallic acid, and 2,4,6-trihydroxybenzaldehyde (THBA), have also been reported to possess various health-promoting properties, such as antioxidant and anti-inflammatory effects [97]. It has been demonstrated that berry intake exerts antimicrobial, antifungal, and antiviral properties by counteracting biofilm formation and contributing to reduced inflammatory response [98]. However, when investigating the effects of anthocyanins on CV health, it is important to consider that after ingestion, polyphenols are rarely detected in serum, with just 1–2% maintaining their original structure [99], as they are metabolized by the gut microbiota and liver in other bioactive components [100]. Indeed, berry-derived polyphenols are generally hydrophilic molecules, making membrane-crossing very challenging. It has been observed that reducing the hydroxyl group increases hydrophobicity for membrane vesicles [101], a metabolic reaction that can be performed by the microbiota, producing completely dehydroxylated metabolites [102,103]. In the gut, anthocyanins are degraded as free anthocyanidins and PCA [104]. Other catabolites derived by anthocyanins include 3-O-methylgallic acid, Gal, SA, VA, and THBA [21,105]. Many of these have been widely studied and shown different beneficial effects on cardiovascular health and gut microbiota composition. Indeed, it has been observed that PCA can reduce miR-10b, increase ATP-binding cassette protein A1 and ATP-binding cassette protein G1 expression [106], and influence microbiota composition, reducing the genera *Prevotella*, *Holdemanella*, and *Ruminococcus* and increasing the *Roseburia* and *Desulfovibrio* genera [107]. Another important metabolite is Gal, which can increase NO levels via the phosphorylation of eNOS [108]. Furthermore, in a study conducted in spontaneously hypertensive rats, Gal inhibited angiotensin-I converting enzyme, leading to reduced BP, comparable to captopril [109]. Another study conducted by Clark and colleagues investigated the role of Gal in atherosclerosis using ApoE −/− mice. Results showed that Gal reduced plaque and led to the disappearance of *Eubacterium fissicatena* induced by high-fat diet in male but not in female mice [110]. Syringic acid is known for its antioxidant, anti-inflammatory, and anti-endotoxic properties [111]. Different in vivo and in vitro studies investigated the effects of SA on cardiovascular health, demonstrating that SA is efficient in reducing cardiac hypertrophic indices, inflammatory markers, and oxidative stress [112] and in modulating NOS production and vasodilation via the activation of the PI3K/Akt/GSK-3b signaling pathway [113]. Furthermore, in vitro gut microbiota fermentation has shown that SA is strongly positively correlated with the increase of the *Bifidobacterium* genus [114]. Similarly, VA is famous for its ability to scavenge ROS, and it is correlated with different pharmacological activities such as antimicrobial, antifungal, antimutagen, hepatoprotective, and cardioprotective effects, to name a few [115]. Concerning this last aspect, it has been observed that treatment with VA results in an upregulation of eNOS expression and a downregulation of endothelin-1 [116]. Furthermore, VA has been shown to exert its cardioprotective effects by modulating AMPK signaling pathways and upregulating endogenous antioxidant markers such as superoxide dismutase, catalase, glutathione peroxidase, and total antioxidant capacity [115,117,118], while an in vitro study conducted on H9c2 cell line showed that VA protection versus H_2_O_2_ damage could be mediated via the regulation of PINK1/Parkin/Mfn2 signaling pathway [119]. Moreover, VA treatment showed anti-inflammatory properties by reducing proinflammatory cytokines, particularly IL-1β, IL-6, and TNF-α [117], and modifying microbiota composition, restoring the relative abundance of *Lactobacillus* and decreasing *Bacteroides* genera [120]. Considering the aforementioned findings, it is evident that anthocyanins and their metabolites have a significant impact on cardiovascular health, both directly and indirectly, via the modulation of gut microbiota composition. However, it is crucial to note that the bioavailability and biotransformation of anthocyanins can be influenced by various factors such as the individuals’ genetic background, age, and lifestyle choices [100]. Moreover, interindividual variability in gut microbiota composition can also affect the metabolism of anthocyanins and the production of bioactive metabolites, resulting in different responses to anthocyanin-containing foods and supplements [121]. Therefore, further research is needed to elucidate the precise mechanisms underlying the cardioprotective effects of anthocyanins and their metabolites, as well as to determine the optimal dosage and duration of anthocyanin intake for maximum cardiovascular benefits. Additionally, a deeper understanding of the complex interplay between anthocyanins, gut microbiota, and host metabolism is required to develop personalized nutritional strategies aimed at preventing and managing CVDs. In conclusion, current evidence suggests that anthocyanins and their metabolites play a crucial role in promoting cardiovascular health via various mechanisms, including the modulation of gene expression, regulation of inflammatory responses, enhancement of endothelial function, reduction of oxidative stress, and alteration of gut microbiota composition. However, the exact molecular pathways and the interindividual variability in anthocyanin metabolism and gut microbiota composition require further investigation. Future research should focus on exploring the personalized effects of anthocyanin-rich foods and supplements as well as developing targeted interventions for the prevention and treatment of CVDs.

## 8. Strengths and Limitations of Existing Evidence

In this narrative review, the main results from clinical studies investigating the effects of anthocyanins on vascular and endothelial outcomes were investigated. Most studies reported significant effects on both vascular and endothelial outcomes, although a non-negligible number of studies showed no significant results. Several previous meta-analyses of individual food sources of anthocyanins, such as pomegranate juice [122] and berries [123], specifically strawberries [124] and chokeberries [125], showed that supplementation was a safe and effective intervention to reduce cardiovascular risk by affecting BP and reducing inflammatory biomarkers and endothelial function [126,127]. However, the evidence of the body of literature reviewed should be considered in light of some limitations. Clinical RCTs on this matter are quite heterogeneous in terms of exposure, number of participants, target population, and doses administered. Moreover, though significant, some results have limited clinical relevance and perhaps would better apply when considered during long-term administration or in pathological conditions. Potential challenges for nutraceutical application of polyphenols include the large variety of pharmacodynamic and pharmacokinetic properties of these molecules that make it difficult to optimize univocal preparations and formulations starting from food sources containing several different molecules (which, in fact, may also have cardioprotective effects, thus confounding the results). Other technical limitations may depend on the scarce bioavailability of certain classes of polyphenols, alteration in polyphenol content after food processing, the important transformation in the colonic gut into metabolites, and potential limitations in reaching the target cells.

## 9. Conclusions

In conclusion, significant effects have been observed for anthocyanins even if substantial heterogeneity in terms of exposure, target population, and doses administered makes it difficult to provide a conclusive opinion on their actual efficacy on BP and other vascular and endothelial outcomes. Further studies are needed to better identify the optimal dosages and formulations for an actual delivery of molecules of interest into the target tissues. Future studies should not only focus on the efficacy of the intervention but should also explore the source of the interindividual heterogeneity of the effects.

## Figures and Tables

**Table 1:** Main characteristic of the clinical trials concerning anthocyanins and cardiovascular risk factors.

Author, Year, Country	Study Design	Participants (Mean Age)	Duration	Treatment	Polyphenol Constituent (Daily Intake)	Comparison	Main Findings
Okamoto, 2020, Japan [25]	Double-blind crossover, RCT	14 adults (73 y)	2 × 7 d (4 wk washout)	600 mg/day New Zealand blackcurrant	210 mg anthocyanins	Placebo containing microcrystalline cellulose	Carotid-femoral PWV (*p* = 0.03) and central BP (*p* = 0.02), brachial SBP (*p* = 0.03), DBP (*p* = 0.02), MBP (*p* = 0.01) and AIX (*p* = 0.03) decreased after the intervention.
Erlund, 2008, Finland [26]	Single-blind, placebo-controlled, RCT	71 individuals with mild hypertension, elevated blood glucose, serum total cholesterol or triacylglycerol and low HDL cholesterol (58 y)	8 wk	2 portions of berries daily (including bilberries, ligonberries, black currant and strawberry purée, chokeberry and raspberry juice)	275 mg anthocyanins (bilberries), 24 mg anthocyanins (ligonberries), 96 mg anthocyanins (black currant and strawberry purée), 120 mg anthocyanins (chokeberry and raspberry juice)	Control products (2 dL sugar-water, 100 g sweet semolina porridge, 100 g sweet rice porridge, and 40 g marmalade sweets)	A significant decrease in mean SBP (*p* = 0.024) in the intervention group in subjects with higher BP at baseline and a significant increase in CADP-CT (*p* = 0.018).
Basu, 2010, USA [27]	Single-blind, controlled, RCT	48 individuals with MetS (50 y)	8 wk	50 g freeze-dried blueberry	742 mg anthocyanins	960 mL/day water	A significant reduction in SBP (*p* = 0.003) and DBP (*p* = 0.04) was reported in the blueberry group.
Curtis, 2019, USA [28]	Double-blind, placebo-controlled parallel, RCT	115 individuals with MetS (63 y)	6 mo	(i) 1 cup blueberries; (ii) 1/2 cup blueberries	(i) 364 mg anthocyanins; (ii) 182 mg anthocyanins	Placebo	A significant improvement was reported in FMD (*p* = 0.003) and systemic arterial stiffness (*p* = 0.04) after the daily intake of 1 cup of blueberries.
Schell, 2019, USA [29]	Crossover, RCT	22 adults with elevated waist circumference and diabetes (54 y)	2 × 4 wk (2 wk washout)	250 g/day frozen red raspberries	225 mg anthocyanins	Control	A decreasing trend in SBP was reported in the raspberry group compared to the control group (*p* < 0.1).
Kimble, 2021, UK [30]	Double-blind, placebo-controlled parallel, RCT	23 healthy volunteers (tart cherry group: 24.7 y; placebo group: 22 y)	4 wk	60 mL/day Montmorency tart cherry	73.6 mg anthocyanins	Placebo	Intervention had no significant effects on SBP and DBP compared to placebo.
Siasos, 2014, Greece [31]	Double-blind, placebo-controlled, crossover, RCT	26 healthy smokers (26 y)	2 × 2 wk (4 wk washout) period	7 cc/kg/day CGJ	472.8 mg total polyphenols, anthocyanins 296 μmol/L in 240 mL of CGJ	Grapefruit juice without polyphenols	CGJ significantly improved FMD (*p* = 0.02) and PWV (*p* = 0.04).
Khan, 2014, UK [32]	Double-blind, placebo-controlled, parallel, RCT	64 participants (52 y)	6 wk	(i) 1000 mL/day low blackcurrant juice drink; (ii) 1000 mL/day high blackcurrant juice drink	(i) 273 mg total polyphenols, 40 mg anthocyanins; (ii) 815 mg total polyphenols, 142 mg anthocyanins	1000 mL/day Placebo	Higher doses led to a significant increase in FMD compared to the placebo group (*p* = 0.022).
Loo, 2016, Finland [33]	Single-blind crossover, intervention, RCT	37 subjects with a SBP of 130–159 mmHg or a DBP of 85–99 mmHg, (55 y)	2 × 8 wk (no washout)	300 mL/day cold-pressed 100% chokeberry juice and 3 g/day oven-dried chokeberry powder	Mean daily intake from the chokeberry products (juice and powder): 2194 mg polyphenols, 1024 mg anthocyanins	300 mL/day placebo juice and 3 g/day powder products	Treatment decreased the daytime ambulatory DBP (*p* = 0.02), and tended to decrease the 24-h DBP (*p* = 0.084) and the true awake ambulatory SBP (*p* = 0.077) and DBP (*p* = 0.057).
Whyte, 2018, UK [34]	Double-blind, placebo-controlled, RCT	112 healthy volunteers (70 y)	6 mo	(i) 1000 mg wild blueberry powder; (ii) 500 mg wild blueberry powder; (iii) 111 mg wild blueberry extract	(i) 2.7 mg anthocyanins; (ii) 1.35 mg anthocyanin; (iii) 7 mg anthocyanins	Placebo containing maltodextrin	A significant reduction in SBP compared to the placebo group (*p* = 0.039).
Chai, 2018, USA [35]	RCT	34 participants (17 men and 17 women) (72 y)	12 wk	480 mL tart cherry juice	450.6 mg total phenolics (gallic acid equivalent), 95.9 mg total tannins	Control drink	Treated group showed a reduced SBP compared to the control group (*p* = 0.04).
Li, 2020, UK [36]	Single-blind crossover, RCT	15 healthy participants (28 y)	2 × 2 wk (1 wk washout)	400 mL/day blood orange juice	960 mg anthocyanins	Control drink	A significant increase in FMD compared to the control drink (*p* = 0.001).
Lynn, 2014, UK [37]	Open-label, placebo-controlled, RCT	47 healthy adults (40 y)	6 wk	250 mL/day cherry drink	273.5 mg total anthocyanins	250 mL/day placebo	No effects were found after treatment.
Lamport, 2016, UK [38]	Double-blind crossover, RCT	19 healthy mothers of preteen children employed for ≥30 h/wk (45 y)	2 × 12 wk (4 wk washout)	355 mL/day CGJ	777 mg total polyphenols, 167 mg anthocyanins as malvidin equivalent, 334 mg proanthocyanidins as catechin equivalent	Energy, taste, and appearance matched placebo	There were no significant effects of interest for SBP and DBP.
Desai, 2018, UK [39]	Single-blind (blinded to participants), placebo-controlled, crossover, RCT	11 healthy participants (30 y)	20 days	260 mL/day MTCJ	540 mg total anthocyanin	Placebo	No differences were observed after treatment.
Aviram, 2004, Israel [40]	Placebo-controlled RCT	19 atherosclerotic patients with carotid artery stenosis	1 y	50 mL/day pomegranate juice	19.2 mg anthocyanins	Placebo	After 1 year of treatment, common carotid intima-media thickness increased by 9% in the placebo group (*p* < 0.01), whereas in the intervention group, it decreased by up to 30% (*p* < 0.01).
Basu, 2010, USA [41]	Controlled, RCT	27 individuals with MetS (47 y)	8 wk	2 cups strawberry beverage (50 g freeze-dried strawberry)	154 mg anthocyanins	4 cups water/d	A significant decrease in VCAM (*p* < 0.05) was reported compared to control.
Dohadwala, 2011, USA [42]	Double-blind crossover RCT	44 overweight individuals with high prevalence of risk factors (juice first: 61 y; placebo first: 63 y)	2 × 4 wk (2 wk washout)	480 mL/day double-strength cranberry juice	94 mg anthocyanins	Placebo beverage not containing polyphenols	No significant results were detected after supplementation
Ruel, 2013, Canada [43]	Placebo-controlled, double-blind crossover, RCT	35 sedentary and healthy overweight men (n = 13 MetS+ and n = 22 MetS−) (45 y)	2 × 4 wk (4 wk washout)	500 mL/day CJC	400 mg total polyphenols, 20.8 mg anthocyanins	500 mL PJ	No differences were observed in AIX between treatments. Significant differences within the groups were observed in AIX (*p* = 0.027) and its response to salbutamol (*p* = 0.04); a significant change in global endothelial function in the CJC group (*p* = 0.02).
Amani, 2014, Iran [44]	Double-blind, controlled, RCTl	36 participants with T2D (51 y)	6 wk	FDS beverage	2006 mg total phenols, 154 mg total anthocyanins	Placebo drink	DBP was significantly reduced in the FDS group compared to placebo (*p* = 0.01).
Asgary, 2014, Iran [45]	Single-blind RCT	21 hypertensive participants (48 y)	2 wk	150 mL/day pomegranate juice	8.7 mg anthocyanins	150 mL water	A significant reduction in SBP (*p* = 0.008), DBP (*p* = 0.046), VCAM-1 (*p* = 0.008) and a significant increase in FMD (*p* = 0.034) compared to baseline.
Tjelle, 2015, Norway [46]	Double-blind, placebo-controlled, RCT	130 healthy individuals with high normal range BP (130/85–139/89 mmHg) or stage 1–2 hypertension (140/90–179/109 mmHg) (62 y)	12 wk	(i) 500 mL/day polyphenol-rich juice based on red grapes, cherries, chokeberries and bilberries; (ii) 500 mL/day similar juice with polyphenol-rich extracts from blackcurrant press-residue	(i) 245.5 mg/100 g polyphenols, 11.9 mg/100 g anthocyanins (ii) 305.2 mg/100 g polyphenols, 41.3 mg/100 g anthocyanin	500 mL/day placebo	A significant decrease in SBP was more pronounced in the hypertensive subjects when analyzed separately (*p* = 0.04). The variation in the BP measurements was significantly reduced in the pooled juice group compared with the placebo group (*p* = 0.03).
Kent, 2017, Australia [47]	Controlled, RCT	42 older adults with mild-to moderate dementia (Alzheimer’s type )(+70 y)	12 wk	200 mL/day cherry juice	138 mg red pigment (anthocyanin)	200 mL/day commercially prepared apple juice	Cherry juice consumption determined a significant reduction in SBP (*p* = 0.038) and a trend for DBP (*p* = 0.160) reduction.
Moazzen and Alizaden, 2017, Iran [48]	Double-blind crossover, RCT	30 patients with metabolic syndrome (51 y)	2 × 1 wk (1 wk washout)	500 mL/day of pure pomegranate juice	50.26 mg anthocyanins, 34.5 mg total phenolics, 119.02 mg total flavonoids	500 mL/day placebo	Pomegranate juice consumption lead to a significant decrease in SBP (*p* < 0.0001) and DBP (*p* = 0.02).
Stull, 2010, USA [49]	Double-blind, placebo-controlled, RCT	32 obese, nondiabetic and insulin-resistant individuals (blueberry group: 54 y; placebo group: 49 y)	6 wk	Smoothie containing 45 g blueberry bioactives	668 mg anthocyanins	Placebo not containing polyphenols	No significant findings were observed in SBP and DBP in the blueberry group.
Basu, 2011, USA [50]	Double-blind, placebo-controlled, RCT	36 individuals with MetS (52 y)	8 wk	480 mL/day cranberry juice	24.8 mg anthocyanins	Placebo juice	No significant changes were observed after treatment.
Krikorian, 2012, USA [51]	Double-blind, placebo-controlled, RCT	21 participants (76 y)	16 wk	Grape juice	(i) 742.3 mg total polyphenols, 150.9 mg anthocyanins, 315.2 mg proanthocyanidins; (ii) 928.4 mg total polyphenols, 188.7 mg anthocyanins, 394.3 mg proanthocyanidins; (iii) 1112.4 mg total polyphenols, 226.1 mg anthocyanins, 472.4 mg proanthocyanidins; (iv) 1298.5 mg total polyphenols, 263.9 mg anthocyanins, 551.5 mg proanthocyanidins	Placebo beverage	No differences in BP were observed in the treated group.
Flammer, 2013, USA [52]	Double-blind, controlled, RCT	69 participants with peripheral endothelial dysfunction and cardiovascular risk factors (49 y)	4 mo	460 mL/day of cranberry juice	800.4 mg total phenolic, 69.46 mg anthocyanins, 1224.52 mg proanthocyanidins	460 mL/day isocaloric placebo juice	Significant reduction in OCN positive EPC (*p* = 0.019).
Basu, 2014, USA [53]	Dose-response, controlled, RCT	60 volunteers with abdominal adiposity and elevated serum lipids (49 y)	12 wk	(i) high-dose freeze-dried strawberry beverage; (ii) low-dose freeze-dried strawberry beverage	(i) 155 mg anthocyanins; (ii) 78 mg anthocyanins	Control beverages	No significant differences were noted in SBP and DBP.
Novotny, 2015, USA [54]	Double-blind, placebo-controlled, parallel, RCT	56 volunteers (50 y)	8 wk	480 mL/day low-calorie cranberry juice	236 mg proanthocyanidins	Placebo beverage	DBP was significantly reduced (*p* < 0.048) in the low-calorie cranberry juice group.
Stote, 2017, USA [55]	Single-blind, placebo-controlled, crossover, RCT	19 women at risk of T2DM (53 y)	2 × 1 wk (8 d washout)	240 mL/day wild blueberry juice	314 mg anthocyanins	Placebo beverage	No significant results in BP were reported. However, a significant increase of NO index production was observed in the blueberry group compared to the placebo group (*p* = 0.039).
Kojadinovic, 2017, Serbia [56]	RCT	23 women with MetS (50 y)	6 wk	300 mL/day pomegranate juice	881.4 mg total phenolic expressed as gallic acid equivalents, 54.9 mg total flavonoids expressed as quercetin acid equivalents, 6.3 mg total anthocyanins expressed as cyanidin-3-glucoside chloride equivalents	Glass of water	Pomegranate juice consumption showed no significant tendency to decrease SBP.
Hollands, 2018, UK [57]	Open label, two-arm crossover, RCT	41 participants with a waist measurement > 94 cm (men) and > 80 (women) (54 y)	28 d	500 mL blood orange juice	50 mg anthocyanins	500 mL blonde orange juice without anthocyanin	No significant differences were observed after treatment.
Espinosa-Moncada, 2018, Colombia [58]	Double-blind crossover, RCT	40 women with MetS (47 y)	2 × 4 wk (4 wk washout)	200 mL/day agraz nectar	4.66 mg anthocyanins	Placebo	SBP and DBP were not affected by the agraz nectar intervention.
Pokimica, 2019, Serbia [59]	Double-blind, placebo-controlled, parallel, RCT	80 volunteers at cardiovascular risk (41 y)	4 wk	100 mL/day chokeberry juice with different doses of polyphenols	(i) 113.3 mg cyanidin-3-glucoside; (ii) 28.3 mg cyanidin-3-glucoside	Placebo	A non-significant reduction in SBP and DBP, compared to the placebo.
Johnson, 2020, USA [60]	Single-blind, placebo-controlled parallel, RCT	26 participants with MetS (tart cherry group: 29.3 y; control group: 44 y)	12 wk	480 mL/day tart cherry juice	176 mg anthocyanins	480 mL/day calorie-matched placebo-control drink	No significant findings were reported in SBP and DBP in the tart cherry group.
Jiménez, 2008, Spain [61]	Controlled, parallel, RCT	43 non-smoking adults (33 y)	16 wk	7.5 g/day grape antioxidant dietary fiber	60 mg anthocyanins	Control	A significant reduction in SBP and DBP was observed in the intervention group (*p* < 0.05).
Ras, 2013, Netherland [62]	Double-blind, placebo-controlled, parallel-group, RCT	70 male and postmenopausal women (63 y)	8 wk	GSE capsule	Monomeric procyanidins (4.3%), dimeric procyanidins (6.1%), trimeric procyanidins (2.5%), gallic acid (4.8%)	Placebo capsule	Significant reduction in SBP and DBP compared to baseline but not versus placebo (*p* < 0.01).
Terauchi, 2014, Japan [63]	Double-blind, placebo-controlled, RCT	91 women with at least one menopausal symptom (~50 y)	8 wk	GSE extract with different doses of proanthocyanidins	(i) 200 mg proanthocyanidins; (ii) 100 mg proanthocyanidins	Placebo	At the end of the trial, SBP and DBP decreased in low-dose and high-dose groups (*p* < 0.001).
Istas, 2019, UK [64]	Double-blind, placebo-controlled parallel, RCT	66 healthy men (24 y)	12 wk	(i) aronia extract capsules; (ii) aronia whole fruit capsules	(i) 30 mg anthocyanins; (ii) 3.6 mg anthocyanins	Placebo containing maltodextrin	A significant increase in FMD was detected after the treatment compared to the placebo group (*p* = 0.0001).
Ahles, 2020, The Netherlands [65]	Double-blind, placebo-controlled parallel, RCT	101 healthy individuals (53 y)	24 wk	(i) 150 mg/day aronia Melanocarpa extract; (i) 90 mg/day aronia Melanocarpa extract	(i) 27 mg anthocyanins; (ii) 16 mg anthocyanins	Placebo containing maltodextrin	A significant reduction in brachial DBP was observed in subjects who received 150 mg/day of the extract (*p* = 0.025).
Hansen, 2005, Denmark [66]	Double-blind, parallel, four-armed, placebo-controlled, RCT	69 healthy subjects (56 y)	4 wk	(i) red wine (M: 300 mL, F: 200 mL); (ii) water + red grape extract (wine-equivalent dose of total polyphenol); (iii) water + red grape extract (half dose)	(i) F: 58 mg anthocyanins; M: 87 mg anthocyanins; (ii) F: 48 mg anthocyanins; M: 71 mg anthocyanins; (iii) F: 24 mg anthocyanins; M: 36 mg anthocyanins	Placebo not containing polyphenols	No significant changes were reported in terms of SBP and DBP among the groups.
Cook, 2015, UK [67]	Double-blind crossover, RCT	14 healthy men trained cyclists (38 y)	2 × 7 d (14 d washout)	300 mg/day NZBC extract	105 mg anthocyanins	300 mg/day microcrystalline cellulose	Resting SBP and DBP did not differ between conditions after 7 days of supplementation.
Schell, 2017, USA [68]	Double-blind crossover, RCT	17 adults (age 57 y)	2 × 12 wk (2 wk washout)	100 g/day freeze-dried strawberry powder	3170 mg total polyphenols, 132 mg total anthocyanins, 440 mg ellagic acid, 100 mg phytosterols	Control beverage daily (total polyphenols 150 mg/d)	No differences were observed in BP.
Kimble, 2021, UK [69]	Double-blind, placebo-controlled parallel, RCT	50 adults (48 y)	3 mo	60 mL/day Montmorency tart cherry	68–73.5 mg anthocyanins	Placebo	No significant findings were observed in SBP and DBP among the groups.
Riso, 2013, Italy [70]	Repeated measures, crossover, RCT	18 male (47 y)	2 × 6 wk (6 wk washout)	250 mL/day of WB drink	375 mg anthocyanins, 127.5 mg chlorogenic acid	Placebo drink	No differences were observed after WB intervention.
Thompson, 2017, Australia [71]	Double-blind, placebo-controlled, crossover, RCT	16 healthy individuals (38 y)	2 × 28 d (2 wk washout)	Anthocyanin supplement	320 mg anthocyanins	Placebo capsules	No significant differences were detected in SBP, DBP, or pulse.
Naruszewicz, 2007, Poland [72]	Double-blind, placebo-controlled, parallel, RCT	44 patients who survived myocardial infarction and had received statin therapy for at least 6 mo (66 y)	6 wk	3 × 85 mg/day chokeberry flavonoid extract	21 mg anthocyanins, 42.5 mg monomeric and olygomeric procyanidins	Placebo containing malthodextrin	A significant reduction in SBP, DBP (*p* < 0.000), sVCAM (*p* < 0.009), sICAM (*p* < 0.05) and MCP-1 (*p* < 0.001) was observed after the aronia flavonoid extract consumption.
Barona, 2012, USA [73]	Double-blind crossover, RCT	24 men with MetS (50 y)	2 × 30 d (3 wk washout)	Grape polyphenol powder	266.8 mg total phenols, 188.6 mg flavans, 35.42 mg anthocyanins, 1.42 mg quercetin, 0.12 mg myricetin, 0.15 mg kaempferol, 0.07 mg resveratrol	Placebo	A significant reduction in SBP and of sICAM-1 (*p* < 0.025) was observed. A significant increase in FMD (*p* < 0.01) in grape group compared to placebo was reported.
Johnson, 2015, USA [74]	Double-blind, placebo-controlled, RCT	40 postmenopausal women with pre- and stage 1-hypertension (blueberry: 59 y; placebo: 57 y)	8 wk	22 g/day freeze-dried blueberry powder	185.9 mg phenolics, 103.18 mg anthocyanins	22 g/day control powder with maltodextrin	In the blueberry group, both SBP and DBP were significantly lower (*p* < 0.05 and *p* < 0.01, respectively) compared to baseline levels. There was also a significant (*p* < 0.01) reduction in brachial-ankle PWV from baseline at 8 weeks and a group × time interaction (*p* < 0.05). No changes to PWC and BP were observed in the control group.
Stull, 2015, USA [75]	Double-blind, placebo-controlled RCT	44 adults with MetS (blueberry: 55 y; placebo: 59 y)	6 wk	45 g/day blueberry smoothie with freeze-dried blueberry powder	1547.2 mg total phenolics, 580.6 mg anthocyanins	Placebo smoothie without the blueberry powder	Improvement of endothelial function in the blueberry group compared to placebo (*p* = 0.024) after adjusting for confounding factors.
Feresin, 2017, USA [76]	Double-blind, placebo-controlled, parallel arm, RCT	60 postmenopausal women with pre- and stage 1-hypertension (59 y)	8 wk	(i) 50 g/day of FDSP; (ii) 25 g/day of FDSP	Phenolic acid: ellagic acid (3.15 mg/d), gallic acid (0.10 mg/d), synapic acid (0.10 mg/d), p-coumaric acid (0.07 mg/d), 2-hydroxycinnamic acid (0.05 mg/d), 3,4-dihydrobenzoic acid (0.04 mg/d); Anthocyanins: cyanidin-3-glucoside (2.91 mg/d), pelargonidin-3-glucoside (99.22 mg/d); flavonols: kaempferol (0.09 mg/d), tiruloside (0.19 mg/d), quercetin (0.67 mg/d), quercetin-3-O-glucoside (1.66 mg/d); flavanones: rutin (0.84 mg/d); flavanols: (+)-catechin (6.26 mg/d); proanthocyanidins (procyanidin B1 (7.70 mg/d)	50 g/day placebo	25 g/day of FDSP led to a significant reduction of SBP (*p* = 0.02) and baPWV (*p* = 0.03) and faPWV (*p* = 0.02).
Arevström, 2019, Sweden [77]	Open-label, controlled, RCT	50 subjects with post-myocardial infarction (median age: 68 y)	8 wk	40 g/day bilberry powder	900 mg anthocyanins	Control	A non-significant reduction in SBP, DBP, and HR was reported in the bilberry group compared to the placebo group.
Zhu, 2011, China [78]	Double-blind, placebo-controlled, parallel, RCT	146 hypercholesterolemic participants (52 y)	12 wk	Anthocyanin capsules	320 mg anthocyanin	Placebo capsules	A significant reduction in SBP (*p* < 0.05) and a significant increase in FMD (*p* < 0.001) compared to baseline were reported.
Jeong, 2016, Korea [79]	Double-blind RCT	51 individuals with MetS (47 y)	12 wk	750 mg/day black raspberry	100 mg cyanidin, 0.4 mg pelargonidin, 19.5 mg proanthocyanidins	Placebo	In the black raspberry group, the AIX decreased significantly (*p* < 0.05).
Curtis, 2009, UK [80]	Double-blind, placebo-controlled, parallel, RCT	52 healthy postmenopausal women (58 y)	12 wk	Elderberry extract	500 mg anthocyanins	Placebo	No significant results for BP and pulse rate were found in the treatment group.
Hassellund, 2012, Norway [81]	Double-blind, placebo-controlled, crossover, RCT	27 healthy participants (41 y)	2 × 4 wk (4 wk washout)	Anthocyanin capsules	640 mg anthocyanins	Placebo capsules	No differences were observed between treated and placebo groups.
Davinelli, 2015, Italy [82]	Double-blind, placebo-controlled, RCT	42 overweight volunteer smokers (55 y)	4 wk	3× capsule of extract of maqui berry/day	162 mg anthocyanins	3× capsule of maltodextrin/d	No statistical differences in BP among the different groups; thus, no effect of maqui berry supplementation on BP.
Zhang, 2016, China [83]	Double-blind, placebo-controlled, RCT	146 hypercholesterolemic subjects (55 y)	24 wk	4 anthocyanin capsules/day	320 mg anthocyanins	4 placebo cups/d	No significant changes were observed in SBP and DBP among the groups.
Estévez-Santiago, 2019, Spain [84]	Parallel, RCT	72 post-menopausal women (59 y)	8 mo	Soft capsules containing anthocyanins	60 mg anthocyanins	(i) 6 mg lutein + 2 mg zeaxanthin; (ii) 60 mg anthocyanins + 6 mg lutein + 2 mg zeaxanthin	No significant results were detected in SBP or DBP among the groups.

Abbreviations: AIX (augmentation index); baPWV (brachial-ankle pulse wave velocity); BP (blood pressure); CGJ (Concord grape juice); CJC (cranberry juice cocktail); CADP-CT (the closing time on the platelet function analyzer when ADP and collagen are used as platelet activators); DBP (diastolic blood pressure); EPC (endothelial progenitor cells); F (female); faPWV (femoral-ankle pulse wave velocity); FDS (freeze-dried strawberry); FDSP (freeze-dried strawberry powder); FMD (flow-mediated dilation); GSE (grape seed extract); HR (heart rate); M (male); MetS (metabolic syndrome); mo (month); MPC-1 (Monocyte chemoattractant protein-1); MTCJ (Montmorency tart cherry juice); NO (nitric oxide); NZBC (New Zealand blackcurrant powder); OCN (osteoblastic marker osteocalcin); PWV (pulse wave velocity); RCT (randomized clinical trial); SBP (systolic blood pressure); sICAM (soluble intercellular cell adhesion molecule); sVCAM (soluble vascular cell adhesion molecule); VCAM (vascular cell adhesion molecule); wk (week); y (year).

## Data Availability

Not applicable.

## Abbreviations

(AIX) augmentation index; (BP) blood pressure; (CGJ) concord grape juice; (CVD) cardiovascular disease; (DBP) diastolic blood pressure; (eNOS) endothelial nitric oxide synthase; (FMD) flow-mediated dilation; (Gal) gallic acid; (ICAM-1) intercellular adhesion molecule-1; (IL) interleukin; (MTCJ) Montmorency tart cherry juice; (NF-κB) nuclear factor-kappa B; (NO) nitric oxide; (NOS) nitric oxide synthase; (PKB/Akt) protein kinase B; (PCA) protocatechuic acid; (MCP-1) monocyte-chemoattractant molecule 1; (PWV) pulse wave velocity; (RCT) randomized clinical trials; (ROS) reactive oxygen species; (SA) syringic acid; (SBP) systolic blood pressure; (THBA) 2,4,6-trihydroxybenzaldehyde; (TNF-α) tumor necrosis factor-alpha; (VA) vanillic acid; (VCAM-1) vascular cell adhesion protein-1.
